# Commentary: Intermittent Fasting and *Akkermansia Muciniphila* Potentiate the Antitumor Efficacy of FOLFOX in Colon Cancer

**DOI:** 10.3389/fphar.2022.843133

**Published:** 2022-02-11

**Authors:** Junhong Su, Henri Braat, Auke Verhaar, Maikel Peppelenbosch

**Affiliations:** Department of Gastroenterology and Hepatology, Erasmus MC—University Medical Center Rotterdam, Rotterdam, Netherlands

**Keywords:** intermittent fasting, the gut microbiome, humans, coloretal cancer, translational medicine

*Akkermansia muciniphila*, a commensal bacterium inhabiting the human intestinal tract, attracts substantial attention as a potential beneficial organism. Being the major producer of propionate in the human intestine, it is endowed with anti-inflammatory properties and linked to improved outcomes in a variety of settings, including the prevention of colorectal cancer (Bian et al., 2019; Su et al., 2020) and metabolic disorders (Plovier et al., 2017). This notion is confirmed by the study Huo et al. (2021), recently published in Frontiers in Pharmacology. In this study the author elegantly show that a pharmacotherapeutical modulatory effect of *A. muciniphila* in a colon cancer xenograft model (CCXM), enhancing FOLFOX efficiency against experimental colorectal cancer. The authors found that the antitumor effects of FOLFOX in CCXM mice were associated with increased abundance of *A. muciniphila*. Oral supplementation with *A. muciniphila* remarkably enhanced FOLFOX efficiency against colon cancer. This study provides hopes for patients with colorectal cancer that by modulating *A. muciniphila* levels also clinical outcomes of FOLFOX treatment can be enhanced. For establishing such effects, however, it is essential to come up with credible strategies that are capable of increasing intestinal levels of *A. municiphila*.

In this context it is important to note that the abundance of *A. muciniphila* is significantly decreased not only in colorectal cancer mice, but also in patients with histologically-confirmed adenoma or colorectal cancer (Wang et al., 2020). Likewise, oral supplementation with *A. muciniphila* reduces colitis-associated tumorigenesis in mice through attenuating DNA damage, increasing tumour cell apoptosis and reducing abnormal proliferation, apparently via increasing the number of CD8^+^ cytotoxic T lymphocytes (Wang et al., 2020). More impressively, even a membrane protein from *A. muciniphila* by itself can exerts effects on colon cancer, similar to those observed when using the pasteurised entire bacterium (Wang et al., 2020). These findings are interpreted as to reflect synergic antitumor effects of this commensal bacterium, which apparently involves at least the production of the relevant bacterial metabolites (*i.e*., short chain fatty acids) and downstream pathways (Louis et al., 2014) as well as with the effects elicited by the membrane components of this pathway. The emerging insight into the mechanisms mediating *A. muciniphila* effects in colorectal cancer further prompts the development of strategies aimed at increasing its intestinal levels in the patients involved.

**FIGURE 1:**
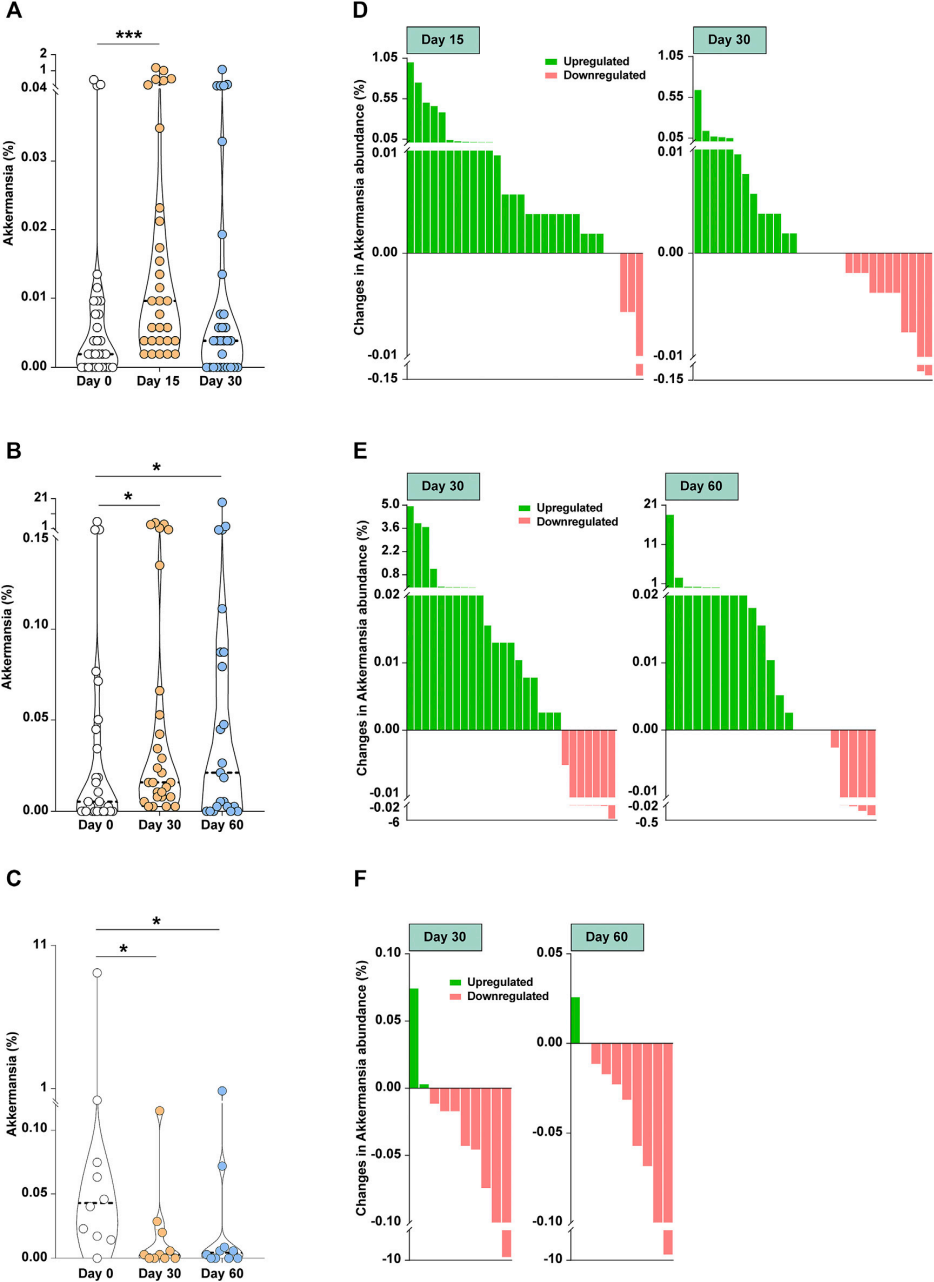
Intermittent fasting upregulated the *Akkermansia municiphila* species abundance in fecal samples obtained in two independent adult cohorts (for details, see reference 6). **(A)** Results showing the dynamics of mean abundance of the bacterium from a cohort of young adult males undergoing intermittent fasting. Fecal samples were collected before (day 0; *n* = 30), during (day 15; *n* = 30) and at the end of intermittent fasting (day 30; *n* = 30). ***p < .001 by Wilcoxon-singed rank test. **(B)** The dynamics of mean abundance of the bacterium from a middle-aged cohort undergoing intermittent fasting. Fecal samples were collected before (day 0; *n* = 27), at the end of (day 30; *n* = 27), and 30 days following the cessation of intermittent fasting (day 60; *n* = 23). *p < 0.05 by Wilcoxon-singed rank test. **(C)** The dynamics of mean abundance of the bacterium from middle-aged unfasted volunteers. Fecal samples were collected at the same time point as in Figure 1B (*n* = 10). *p < 0.05 by Wilcoxon-singed rank test. **(D)** Change in *A. municiphila* at day 15 (left) or day 30 (right) was calculated for each individual in the young cohort by subtracting the relative abundance observed before fasting from that of during or after fasting. As a result, 83.3 percent (25/30) of volunteers displayed upregulated bacterium abundance at day 15 and 43.3 percent at day 30. **(E)** Change in *A. municiphila* at day 30 (left) or day 60 (right) for each individual in the middle-aged cohort was calculated as above. The bacterium was upregulated in 74.1 percent of volunteers (20/27) at day 30 and 60.9 percent (14/23) at day 60. **(F)** Change in *A. municiphila* at day 30 (left) or day 60 (right) was calculated for each unfasted individual as above. The bacterium abundance was increased in only 20 percent of volunteers (2/10) at day 30 and 10 percent (1/10) at day 60 in this group. In the present study, 16S rRNA sequencing was applied to calculate the abundance of *A. municiphila* at each time point.

Unfortunately, the earlier studies by Hou et al. (2021) and Wang et al. (2020) do not address this point directly and further work is necessary to exploit the potential of this bacterium in increasing the efficacy of. FOLFOX. Importantly, we recently conducted a study in volunteers either submitting themselves a month of intermittent fasting (involving approximately 16 h of absence of food intake *per* day) or served as non-fasting controls and characterised the microbiome in these volunteers (Su et al., 2021). Strikingly, among the various effects intermittent fasting exerted on the microbiome we also observed that *A. municiphila* levels were strongly upregulated in the fasting volunteers (Figures 1A,B), *but inversely changed in* unfasted volunteers (Figure 1C). Furthermore, *A. municiphila* was increased in more than 83% individuals in the young cohort (Figure 1D) and 74% in the middle-aged cohort (Figure 1E), possibly as a consequence of the competitive advantage this organism obtains in the absence of food as it can degrade intestinal mucins. However, upregulated in no more than 20% individuals in unfasted group (Figure 1F). These data would fit well recent data that fasting *per se* decreases the propensity to contract colorectal cancer (Weng et al., 2020). Thus in conjunction with the data presented by Hou et al. (2021) it would thus be rational to propose that intermittent fasting for patients who are taking medications such as FOLFOX or patients at risk for colorectal cancer (*e.g.*, patients with inflammatory bowel with a history of neoplasms) would benefit from intermittent fasting.
